# A pilot randomised clinical trial of physiotherapy (manual therapy, exercise, and education) for early-onset hip osteoarthritis post-hip arthroscopy

**DOI:** 10.1186/s40814-017-0157-4

**Published:** 2017-07-07

**Authors:** Joanne Kemp, Kate Moore, Marlene Fransen, Trevor Russell, Matthew Freke, Kay M Crossley

**Affiliations:** 1 La Trobe Sport and Exercise Medicine Research Centre, School of Allied Health, College of Science, Health and Engineering, La Trobe University, Melbourne, VIC Australia; 2 Australian Collaboration for Research into Injury in Sport and its Prevention, Faculty of Health, Federation University Australia, Ballarat, VIC Australia; 3 Bodysystem P/L, 38 Collins St, Hobart, Tasmania Australia; 4 School of Physiotherapy, Faculty of Health Sciences, The University of Sydney, Sydney, NSW Australia; 5 School of Health and Rehabilitation Sciences, Faculty of Health and Behavioural Sciences, University of Queensland, Brisbane, Australia

**Keywords:** Hip arthroscopy, Physiotherapy, Rehabilitation, Randomised controlled trial, Chondropathy, Osteoarthritis

## Abstract

**Background:**

Despite the increasing use of hip arthroscopy for hip pain, there is no level 1 evidence to support physiotherapy rehabilitation programs following this procedure. The aims of this study were to determine (i) what is the feasibility of a randomised controlled trial (RCT) investigating a targeted physiotherapy intervention for early-onset hip osteoarthritis (OA) post-hip arthroscopy? and (ii) what are the within-group treatment effects of the physiotherapy intervention and a health-education control group?

**Methods:**

This study was a pilot single-blind RCT conducted in a private physiotherapy clinic in Hobart, Australia. Patients included 17 volunteers (nine women; age 32 ± 8 years; body mass index = 25.6 ± 5.1 kg/m^2^) who were recruited 4–14 months post-hip arthroscopy, with chondropathy and/or labral pathology at the time of surgery. Interventions included a physiotherapy treatment program that was semi-standardised and consisted of (i) manual therapy; (ii) hip strengthening and functional retraining; and (iii) health education. Control treatment encompassed individualised health education sessions. The primary outcome measure was feasibility, which was reported as percentage of eligible participants enrolled, adherence with the intervention, and losses to follow-up. The research process was evaluated using interviews, and an estimated sample size for a definitive study is offered. Secondary outcomes included the Hip disability and Osteoarthritis Outcome Score (HOOS) and the International Hip Outcome Tool (IHOT-33) patient-reported outcomes.

**Results:**

Seventeen out of 48 eligible patients (35%) were randomised. Adherence to the intervention was 100%, with no losses to follow-up. The estimated sample size for a full-scale RCT was 142 patients. The within-group (95% confidence intervals) change scores for the physiotherapy group were HOOS-Symptoms 6 points (−4 to 16); HOOS-Pain 10 points (−2 to 22); HOOS-Activity of Daily Living 8 points (0 to 16); HOOS-Sport 3 points (−12 to 19); HOOS-Quality of Life 3 points (−7 to 13); and IHOT-33 7 points (−10 to 25). The within-group (95% confidence intervals) change scores for the control group were HOOS-Symptoms −4 points (−17 to 9); HOOS-Pain −2 points (−18 to 13); HOOS-Activity of Daily Living −7 points (−17 to 4); HOOS-Sport 4 points (−16 to 23); HOOS-Quality of Life −5 points (−18 to 9); and IHOT-33 −4 points (−27 to 19). Suggestions to improve study design included greater supervision of exercises and increased access to physiotherapy appointments.

**Conclusions:**

Results support the feasibility of a full-scale RCT, and recommendations for an adequately powered and improved study to determine the efficacy of this physiotherapy intervention post-hip arthroscopy to reduce pain and improve function are provided.

**Trial registration:**

Australian Clinical Trials Registry, ACTRN12614000426684

## Background

Musculoskeletal conditions, including osteoarthritis (OA) of the hip, are an important cause of disability internationally, and the impact of hip OA on young and middle-aged people is significant [1, 2]. The identification of physiotherapy interventions that can reduce the symptomatic progression of hip OA is important and may limit the public health burden seen in this disease [3, 4]. Hip joint pathology is a common cause of hip pain [5, 6] amongst people aged 18–50 years [7, 8] and may represent early hip OA [9]. The increase in hip arthroscopic surgery in countries such as Australia [10] and the USA [11] has increased the diagnosis of hip joint pathology [12]. There have been a number of clinical protocols (level 5 evidence, which is the lowest form of research evidence) [13–15] that have described physiotherapy programs following hip arthroscopy. These protocols describe weight-bearing progression, range of motion exercise, gentle strength exercise, and return to activity [13, 15]. Similarly, level 5 studies have described the development of non-surgical treatment protocols for femoroacetabular impingement [16]. Despite this, there is no level 1 evidence to support physiotherapy rehabilitation programs following hip arthroscopy.

Chondropathy, which may indicate early-onset hip OA, is a common finding in people who undergo hip arthroscopy and is associated with worse pain, difficulty participating in physical activity, and reduced quality of life compared to healthy controls [9]. In addition, people with early-onset hip OA demonstrate reduced hip joint range of motion (ROM) and hip muscle strength compared to healthy controls [17], as well as poor performance and reduced balance on functional tasks such as single-leg squat [18, 19]. Better performance in hip joint ROM and muscle strength is associated with better quality of life outcomes in people with early-onset hip OA after hip arthroscopy [20]. Therefore, interventions that target physical impairments may improve quality of life outcomes in this patient group.

As full-scale randomised controlled trials (RCTs) are expensive, it was important to establish feasibility before undertaking such a study [21]. No RCT examining the effects of a physiotherapy intervention had been undertaken in this population, and therefore, adherence with the intervention, patient and therapist willingness to participate, feasibility of recruitment, and acceptability of interventions were unknown. Therefore, before committing to the expense of a full-scale RCT, an understanding of the feasibility of such a study was required. The primary aims of this pilot study were to determine the feasibility of a future definitive study, estimate the sample size for such a definitive study, and offer recommendations regarding research design. To achieve these aims, the objectives were to

1. Interview participants and practitioners/therapists regarding (a) their willingness to participate in the study and (b) their opinions on the research process
2. Analyse collected data regarding (a) participant enrolment, (b) participant adherence with the intervention, and (c) participant losses to follow-up
3. Estimate the sample size and likely time-frames of recruitment of a future fully powered study
4. Offer a set of recommendations that would support the implementation of a definitive study

The secondary aim was to offer insights with regard to the possible effect of the proposed intervention(s) post-arthroscopy for early hip OA based on the selected measurement outcomes. To achieve this aim, the objectives were to

1. Collect data at specified time-points using the selected outcome measures from the two intervention groups
2. Analyse this data using the appropriate inferential statistical (ANCOVA) and evaluate the change in scores within the two intervention groups

## Methods

The methods for this feasibility study were previously published as a protocol manuscript in detail [22].

### Study design overview

This was a parallel group, randomised, single-blind, controlled clinical trial conforming to SPIRIT [23] guidelines. The trial protocol was registered with the Australian Clinical Trials Registry (ACTR number: 12614000426684). Ethics approval was obtained through the University of Queensland Medical Research Ethics Committee (number: 2013001553). All study participants provided written informed consent.

### Setting and participants

This study was undertaken in a private physiotherapy clinic in Hobart, Tasmania, Australia. Seventeen participants (presented as mean ± standard deviation) (nine female; age = 32 ± 8 years; height = 1.71 ± 0.08 m; weight = 77 ± 17 kg; body mass index = 25.6 ± 5.1 kg/m^2^; time since surgery = 9 ± 3 months) were recruited 4 to 14 months post-hip arthroscopy surgery performed by a single surgeon with extensive expertise in hip arthroscopy. The project investigator (KM) screened participants for eligibility based on history and examination.

#### Inclusion criteria

The inclusion criteria are as follows: (i) aged 18 to 50 years; (ii) arthroscopy for intra-articular hip pathology during the past 4 to 14 months; (iii) evidence of early-onset hip OA (defined as chondropathy Outerbridge grade ≥1) [24] at time of hip arthroscopy which is equivalent to OARSI grade 2 = surface discontinuity [25] and usually not visible on radiographs; and (iv) pain in the hip ≥30/100 mm on a visual analogue scale on aggravating activities.

#### Exclusion criteria

The exclusion criteria are as follows: (i) pain not confirmed by physical examination of the hip [26, 27]; (ii) concurrent symptoms of hip bursitis or tendinitis; (iii) surgical complications including infection; (iv) planned lower limb surgery in the following 12 months; (v) physical inability to weight-bear fully or undertake testing procedures; and (vi) inability to understand written and spoken English.

### Randomisation and interventions

Potential participants were identified by the surgeon and invited to contact the project co-ordinator. Volunteers were then screened via telephone interview to confirm eligibility by the blinded project investigator (KM). Once eligibility was confirmed, the blinded project investigator (KM) then performed outcome assessments at baseline and 3 months. Participants were instructed not to divulge their group allocation to the assessor. Participants were asked to not undertake other treatments with the exception of stable drug doses. Treating physiotherapists recorded treatment as per the protocol [22].

The computer-generated randomisation schedule was produced by the University of Queensland, School of Health and Rehabilitation Sciences. This was revealed via telephone following the completion of the baseline assessment to the project co-ordinator (JK), and the treating physiotherapists were informed of group allocation prior to commencement of the intervention.

The interventions used in this study were described previously in detail [22]. Briefly, participants in both groups were treated by one of three experienced physiotherapists. The treating physiotherapists were trained and proficient in both interventions (physiotherapy and control). The intervention was a face-to-face physiotherapy intervention and was delivered in eight sessions over 12 weeks (once per week for 4 weeks, then once per fortnight for 8 weeks).

#### Physiotherapy intervention

The physiotherapy intervention was a semi-standardised program consisting of (i) manual hip joint and soft tissue mobilisation and stretching; (ii) hip muscle retraining; (iii) trunk muscle retraining; (iv) functional, proprioceptive and sports- or activity- specific retraining; (v) enhancing physical activity; and (vi) education. The physiotherapy intervention was progressed based on response to exercise load, thus maximising the training effects, and included supervised exercises during each visit. In addition, a home exercise program was encouraged to be performed independently four times per week, using a structured exercise manual.

#### Control

The control intervention was delivered by the same treating physiotherapists at the same frequency and duration as the physiotherapy intervention, to control for the psychosocial aspects of face-to-face physiotherapy. The control intervention encompassed individualised health education sessions covering topics such as exercise, diet, weight loss, and appropriate stretching. Participants in the control group were also provided with a treatment manual containing specific education information sheets.

### Outcomes and follow-up

#### Primary outcome measure: feasibility of a full-scale RCT

Feasibility was assessed by evaluating the number of eligible participants randomised, adherence with intervention, and loss to follow-up. In addition, strengths and weaknesses of research process and design and research environment were determined following qualitative interviews with the project manager, treating physiotherapists and included participants to inform future trial design. Finally, feasibility included an estimate of the required sample size for any future, full-scale trial [28].

#### Secondary outcome measure: hip-related signs

Hip-related symptoms were measured with the symptoms and stiffness, pain, activities of daily living, and sports and recreation subscales of the Hip disability and Osteoarthritis Outcome Score (HOOS) [29]. The HOOS subscales are scored from 0–100 points where 100 represents the best possible score. The HOOS has been previously reported as an appropriate PRO for use in a hip arthroscopy population [30].

#### Secondary outcome measures: hip-related QoL

The patient-reported outcome (PRO) measures that measured hip-related quality of life were the International Hip Outcome tool (IHOT-33) and HOOS quality of life subscale (HOOS-Q). The IHOT-33 is a composite score that was developed for specific use in a hip arthroscopy population [31]. It is reliable (intraclass correlation coefficient (ICC) = 0.93; 95% CI 0.87 to 0.96), with a low standard error of measurement of 6 points out of 100, and is valid and responsive, with a minimal important change (MIC) of 10 points [30]. The HOOS-Q [29] is reliable (ICC = 0.95; 95% CI: 0.84 to 0.97), with a low SEM (5/100 points), with a MIC of 11 points [30] in people post-hip arthroscopy. Both measures are scored from 0–100 points, where 100 represents the best possible score.

#### Secondary outcome measures: hip muscle strength and hip joint range

Hip muscle strength and hip joint flexion range were measured using our published methods [18, 32], with high reliability (ICC range from 0.87 to 0.95), and have been outlined in the published study protocol [22]. Briefly, all strength tests were performed with a Commander Power track II (J-Tech medical) hand-held dynamometer as an isometric muscle contraction (i.e. “make” test) [33]. Hip muscle strength was reported as peak torque normalised for bodyweight and was calculated by multiplying the force (measured in Newtons (N)) by the length of the moment arm (measured in metres (m)) and then divided by body weight (measured in kilograms (kg)) (i.e. Nm/kg). Active Hip flexion range of motion was measured using a Plurimeter (Dr Rippstein, Switzerland) inclinometer [18].

### Other measures

Other measures of body anthropometry were collected including weight, height, body mass index, and waist girth.

### Statistical analyses

The primary outcome measure of feasibility was reported as the percentage of eligible participants randomised, percentage of participants adhering with the intervention, and losses to follow-up over the treatment period. For the secondary outcome measures, change scores for each group from baseline to follow-up were determined and reported as mean (95% confidence interval) change score using analysis of covariance (ANCOVA). Co-variates of age and gender were utilised.

Given the very small sample used in this study, it was deemed best to not calculate an effect size to provide a recommendation for estimated sample size for a full-scale RCT, as recommended by Eldridge and colleagues [34]. Instead, the recommended sample size for future studies will be presented based on a standard moderate effect size (0.5) [35] for the outcomes of pain and function with a minimum of 80% power (*α* = 0.05). All statistical analyses were performed using SPSS Version 22.0 software (SPSS Inc., Chicago, IL, USA).

## Results

Baseline characteristics, PROs, and measures of physical impairment for both treatment allocation groups are presented (Table 1).

**Table 1:** Baseline characteristics

Baseline characteristic	Education control group (mean(SD)) *n* = 7	Physiotherapy intervention group (mean(SD)) *n* = 10
Age (years)	31.4(6.4)	31.7(10.0)
Gender (men/women)	2/5	6/4
Time since surgery (months)	8.0(3.6)	9.1(3.4)
Height (metres)	1.71(0.08)	1.72(0.06)
Weight (kilograms)	73(14)	79(19)
BMI (kg/m^2^)	24.4(4.7)	26.3(5.3)
HOOS-Symptoms	61.4(9.9)	66.5(14.9)
HOOS-Pain	76.8(17.4)	69.6(22.5)
HOOS-Activity of Daily Living	86.9(10.9)	80.3(15.9)
HOOS-Sport	78.0(14.0)	68.7(22.0)
HOOS-Quality of Life	51.0(15.5)	49.0(25.0)
IHOT-33	58.1(17.3)	62.6(23.7)
Flexion ROM (°)	90(51)	102(34)
Abduction strength(Nm/kg)	1.31(0.48)	1.51(0.54)
Adduction strength (Nm/kg)	1.20(0.61)	1.29(0.39)
Extension strength (Nm/kg)	1.12(0.55)	1.14(0.63)
Flexion strength (Nm/kg)	1.22(0.55)	1.22(0.52)
ER strength (Nm/kg)	0.69(0.34)	0.67(0.30)
IR strength (Nm/kg)	0.44(0.19)	0.53(0.25)

*SD* standard deviation, *n* number, *HOOS* Hip disability and Osteoarthritis Outcome Score, *IHOT-33* International Hip Outcome Tool, *ROM* range of motion, *ER* external rotation, *IR* internal rotation, *Nm/kg* peak torque normalised for body weight measured in Newton metres per kilogram, *CI* confidence interval

**Fig. 1:**
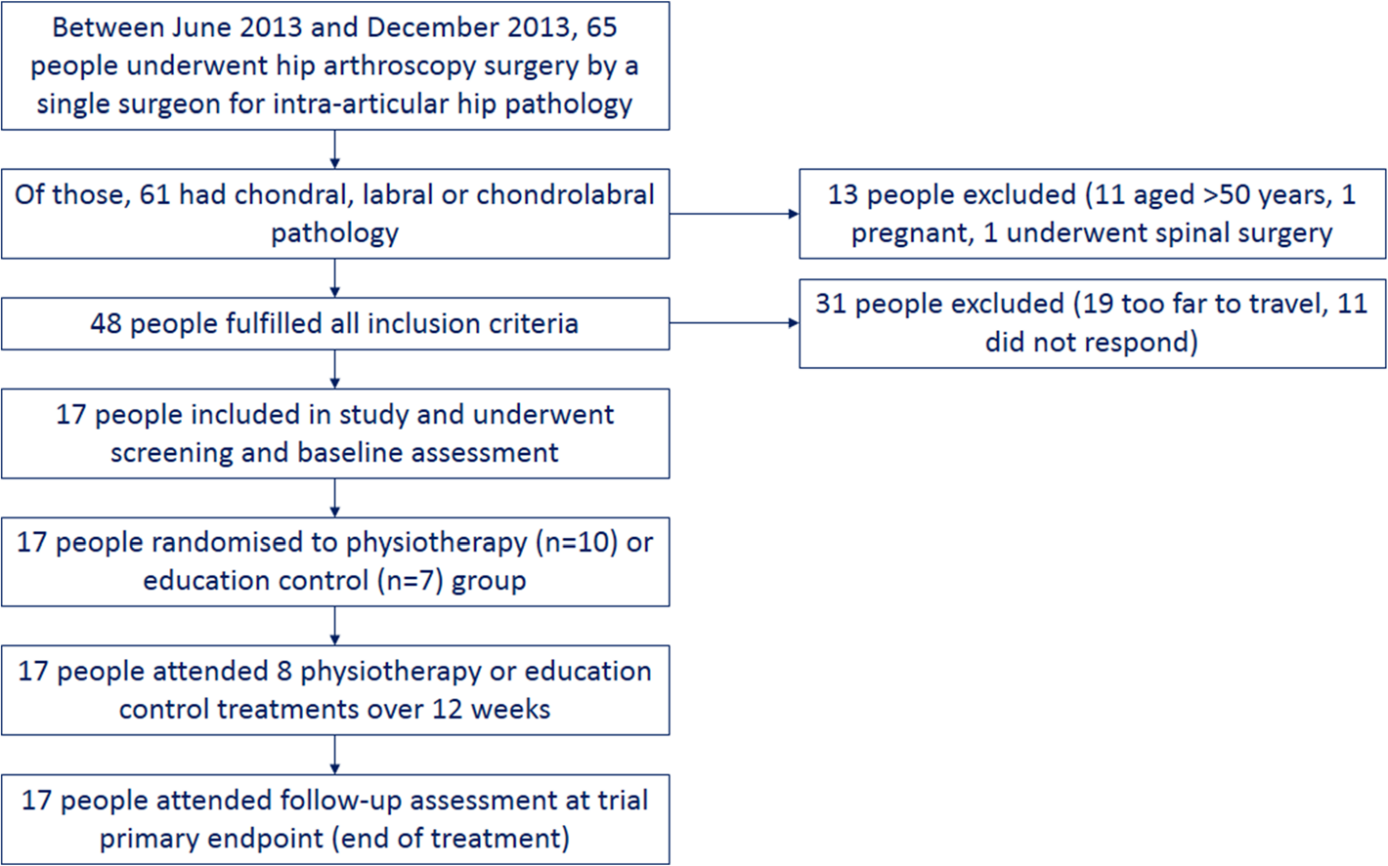
Flowchart of participants through the study

Forty-eight patients who fulfilled all eligibility criteria underwent hip arthroscopy over a 6-month period. Of 48 potentially eligible people fulfilling all inclusion criteria, 17 (35%) participants were randomised over a 4-month period (Fig. 1). Thirty-one people were not willing to participate in the study, including 19 people who responded it was too far to travel and 11 who did not respond to the invitation. The remaining 19 people could not participate due to the distance required to travel for the study treatment. No participants were lost to follow-up, and all participants from both groups completed the eight scheduled treatment sessions. No adverse events were recorded.

For a standardised effect size between the two groups of 0.5 [35] (95% confidence interval), with a minimum of 80% power (*α* = 0.05), 128 participants (64 in each group) are required. For future full-scale RCTs, we would recommend allowing 10% loss to follow-up. Increasing the sample to 142 participants (71 in each group) would allow for this.

Post-study interviews revealed that the participants included in this study expressed a wish to reduce their hip pain and participate in more family activities, postponing joint replacement surgery for as long as possible. They also expressed a willingness to participate in similar trials investigating non-surgical interventions for early hip OA. Physiotherapists involved in this study expressed willingness to take part in further studies and felt valued in being able to contribute to the body of knowledge for best practice management of this condition. Participants from both treatment groups felt that the interventions utilised were credible. Feedback from participants and physiotherapists indicated that future studies would be improved through additional supervision of exercise programs to enhance compliance and motivation, as well as offering a wider range of appointment times to fit with patients’ work and family responsibilities.

Patient-reported outcome change scores (mean, 95% confidence intervals) for each group separately are contained in Table 2. Change scores for each group separately for the impairment measures (mean, 95% confidence intervals) are contained in Table 3.

**Table 2:** Change (between baseline and end of treatment), for patient-reported outcome (PRO) for the education control and physiotherapy groups

PRO measure	Education control group (mean change (95% CI)	Physiotherapy intervention group (mean change (95% CI))
HOOS-Symptoms	−4 (−17 to 9)	6 (−4 to 16)
HOOS-Pain	−2 (−18 to 13)	10 (−2 to 22)
HOOS-Activity of Daily Living	−7 (−17 to 4)	8 (0 to 16)
HOOS-Sport	4 (−16 to 23)	3 (−12 to 19)
HOOS-Quality of Life	−5 (−18 to 9)	3 (−7 to 13)
IHOT-33	−4 (−27 to 19)	7 (−10 to 25)

Covariates included in the model = age, gender. *HOOS* Hip disability and Osteoarthritis Outcome Score, *IHOT-33* International Hip Outcome Tool

**Table 3:** Change for physical impairment measures for the education control and physiotherapy groups

Impairment measure	Education control group (mean change (95% CI))	Physiotherapy intervention group (mean change (95% CI))
Flexion ROM (°)	−1 (−6 to 4)	3 (−1 to 7)
Abduction strength (Nm/kg)	0.3 (−0.1 to 0.6)	0.2 (−0.1 to 0.5)
Adduction strength (Nm/kg)	0.5 (0.1 to 0.9)	0.1 (−0.2 to 0.4)
Extension strength (Nm/kg)	0.4 (0.2 to 0.6)	0.4 (0.3 to 0.6)
Flexion strength (Nm/kg)	0.4 (0.1 to 0.6)	0.3 (0.1 to 0.5)
ER strength (Nm/kg)	0.2 (0.0 to 0.3)	0.1 (0.0 to 0.3)
IR strength (Nm/kg)	0.1 (0.0 to 0.3)	0.0 (−0.1 to 0.1)

Covariates included in the model = age, gender. *ROM* range of motion, *ER* external rotation, *IR* internal rotation, *Nm/kg* peak torque normalised for body weight measured in Newton metres per kilogram, *CI* confidence interval

## Discussion

The results of this study suggest a full-scale RCT is feasible. Thirty-five percent of eligible participants were randomised; there was excellent study treatment adherence and no loss to follow-up at the end of the study treatment period. Participants and treating physiotherapists expressed a willingness to take part in future studies and offered suggestions for improvement. An estimated sample of 142 participants would be required in a full-scale RCT.

The successful completion of a RCT evaluating a physical intervention faces a number of challenges. Clinician barriers include time constraints, lack of staff training for treating therapists, concern for patients’ welfare, loss of professional autonomy, difficulty with the consent procedure, and lack of rewards and recognition. Patient barriers include additional time demands of taking part in a trial for the baseline and follow-up assessment and concerns about information and consent [36]. Funding constraints meant that the number of intervention sessions was capped at eight visits regardless of symptoms and constrained to 12 weeks of duration during a late-stage period of rehabilitation when the longer-term gains from manual therapies may have plateaued. Based on feedback from participants, greater availability of appointments and increased number of supervised exercise sessions would improve motivation and compliance. In order to achieve this, additional funding is likely to be required to support a full-scale study.

The rate of recruitment in this feasibility study was modest, with 17 participants out of 48 (35%) prospective subjects participating. While many studies do not report their rate of recruitment, other RCTs examining physiotherapy intervention cite from 32% for OA total knee joint replacement [37] and 62% for OA hip [38]. Full RCTs examining physiotherapy treatment effects on musculoskeletal conditions have utilised much larger sample sizes designed to detect statistically significant changes based on the MCI of the measuring tool used as well as allowing for a 10–15% drop out rate [39, 40]. The results from this study suggest that a sample of 142 participants would be required, allowing for 10% loss to follow up. To progress this study to a full-scale RCT, with recruitment occurring over 2 years, 405 eligible patients would need to be invited to take part to achieve adequate numbers from 35% recruitment rate. As such, the number of participating surgeons would need to be increased to recruit adequate numbers within an acceptable trial period. For this study, we recruited five patients per month from a single surgeon and single physiotherapy centre. If a full-scale RCT was conducted in a single centre, recruitment would take 28 months. If the number of surgeons and physiotherapy centres was doubled, we estimate recruitment into a full-scale RCT would be achieved in 14 months. Additional recruitment and retention incentives could be employed to enhance these aspects of a full-scale RCT.

Participants recruited into this study were aged 31 years on average, with a normal to slightly overweight BMI. We used inclusion criteria for early-onset hip OA as chondropathy greater than or equal to Outerbridge grade 1 [24], which is equivalent to OARSI grade 2 (chondral surface continuation) [25]. This stage of OA is generally not visible on radiographs, and therefore, our patients are younger than those with early to moderate hip OA using radiographic OA grading systems such as the Kellgren and Lawrence scale [41]. Even taking these differences into consideration, it is possible that participants in future studies, and patients seen in clinical practice, will be older, with greater BMI and more advanced hip OA than those included in this study. In an older, more overweight group, with more advanced OA, improvements in outcomes and differences between groups may be smaller. As such, in older patients, a larger sample size than the 106 people recommended in this study may be required. Future studies may choose to stratify groups based on factors such as age or treatment centre, and the required sample size would need to be increased to reflect this. Greater improvements in PRO in this feasibility study are promising for physiotherapy rehabilitation intervention, and this supports a larger study, with recommended participant numbers (*n* = 142) to detect a between-group difference in change scores.

Flexion ROM did not improve in either group. Improving hip flexion was a target of the evaluated physiotherapy intervention/treatment since greater flexion ROM was independently associated with better scores in both HOOS-Q and IHOT-33 12 to 24 months post-hip arthroscopy [20]. It is unclear why flexion ROM did not improve in the current study. It is possible that the patients in this study, who were up to 18 months post-hip arthroscopy, may have reached the ceiling in their potential hip flexion ROM [20]. The current study was also most likely underpowered to detect a statistically significant change in ROM. Clinical guidelines and anecdotal evidence suggests that clinician-administered mobilisations might be effective in patients with hip pain, but there is little evidence to support this [42]. Future studies with larger patient groups that have adequate power to detect an improvement are required.

Muscle strength gains were minimal in the current study. Improving muscle strength was a key component of the physiotherapy intervention. The training program was based on the best available knowledge of impairments in people post-hip arthroscopy [40, 43–45]. The number of sets and repetitions were deemed adequate to introduce changes in the muscular system in previously untrained subjects [46], and strength gains were expected within a 12-week time period [47]. The lack of measured muscle strength change in the intervention group may be due to a number of factors: (i) the specificity of the strength exercises, while improving functional ability, may not have transferred to improved strength using open-chain, isometric testing. ‘Transfer of training’ is a term used to describe the effectiveness of adaptations from a strength exercise transferring to functional performance [48]. In this study, testing force production in an isolated manner may have reduced the validity of the overall force capabilities of the participant’s leg musculature [47]. (ii) It is possible that more marked strength, coordination, and neural adaption gains may have already occurred during earlier phases of rehabilitation, prior to patients entering this study [49, 50]. A systematic review of rehabilitation following hip arthroscopy [51] categorises the rehabilitation phase from 3 to 7 months post operation as ‘return to sport’ meaning that for the recruits in this study, most of these neural adaptions may have already occurred. (iii) Progressive overload of the exercise is necessary for maximal muscle fibre recruitment, and consequently, muscle fibre hypertrophy and strength increases [50]. Variations in load, length or volume of training programmes affect the rate of strength gain. It is possible that while the exercises in this study contained progressive resistance, they were not optimally progressed or were too easy in terms of repetitions and load to induce hypertrophy and strength change. (iv) Numerous resistance-training studies have shown frequencies of 2–3 alternating days per week in previously untrained individuals to be an effective initial frequency whereas 1–2 days per week appear to be an effective maintenance frequency for those individuals already engaged in a resistance training program [52]. The frequency and compliance to frequency of exercise in this study may have been too low to optimise strength gains. (v) Finally, while reported compliance was good, it is possible that participants did not fully engage with the programme when outside the supervision of the treating physiotherapists. Future studies that are adequately powered need to utilise current knowledge in strength and conditioning and surrounding technologies to optimise the opportunity for strength gains. Exercise programs should be commenced earlier; there should be greater incentives for compliance, with more focus on monitoring home exercises; treating therapists should more actively monitor the progression of exercise program, possibly with physiotherapist-supervised exercise sessions. If future studies include participants who are older and heavier, the length of the intervention may need to be increased to accommodate the likely slower gains in strength expected.

As well as addressing the criteria of a high quality RCT, this study had a number of strengths including the willingness of the study population to remain in the trial and use of the same practitioners in the same environment for both treatment groups to minimise sources of variation. The PRO measures used have been demonstrated previously to have the best psychometric properties for use in a post-hip arthroscopy cohort. [30] Additionally, the measurement tools for ROM, strength, and function are reliable, with predetermined MIC levels. The majority of potential limitations of this study have been outlined above. As no strength changes were observed, it is possible that the duration, content, and load variables of the exercise programme may not have been optimal. In addition, this study did not collect information on adherence to the home exercise program. Further, we did not determine acceptable thresholds of feasibility a priori; instead, we chose to explore these aspects to inform the design of future, fully powered RCTs. It is important that these results are not interpreted as definitively supporting one intervention over the other, given the very small sample used in this study. Finally, the participants in the education control group were unblinded and were aware with their treatment group allocation, given the differences in treatments. All efforts were made to minimise the impact of this through the same treatment location, frequency, and duration of treatment applying to both groups. In addition, the same treating physiotherapists treated patients from each group. However, this may have resulted in more negative follow-up scores in the control group. Future full-scale RCTs may choose a control intervention that more closely resembles the physiotherapy intervention. These limitations should be considered from the viewpoint that the aim of a feasibility study is to determine the viability of continuing with an adequately powered RCT and identify potential improvements to the study protocol and procedures.

## Conclusions

Feasibility results support a full-scale RCT to determine the efficacy of physiotherapy interventions in people with early hip OA. Participants were willing to take part and offered suggestions for study improvements. For a full-scale RCT, at least 142 participants would need inclusion. In addition, we recommend a number of modifications to the intervention to enhance its effectiveness. Timely recruitment would be enhanced by using at least two surgeons and physiotherapy centres in a full-scale study. This study provided encouraging results for the physiotherapy intervention in the areas of improved PROs (iHOT-33 and HOOS) affecting symptoms, pain, and activities of daily living. Flexion ROM was also improved by a minimal amount in the physiotherapy intervention group while muscle strength did not change. The results of this feasibility trial suggest that a full-scale RCT with participant numbers appropriate to achieve statistical power and strength programme modification is warranted.
